# Multivariable regression analysis of febrile neutropenia occurrence in early breast cancer patients receiving chemotherapy assessing patient-related, chemotherapy-related and genetic risk factors

**DOI:** 10.1186/1471-2407-14-201

**Published:** 2014-03-19

**Authors:** Alena M Pfeil, Christof Vulsteke, Robert Paridaens, Anne-Sophie Dieudonné, Ruth Pettengell, Sigrid Hatse, Patrick Neven, Diether Lambrechts, Thomas D Szucs, Matthias Schwenkglenks, Hans Wildiers

**Affiliations:** 1 Institute of Pharmaceutical Medicine (ECPM), University of Basel, Basel, Switzerland; 2 Department of Oncology, Laboratory of Experimental Oncology (LEO), KU Leuven, Leuven, Belgium; 3 Department of General Medical Oncology, University Hospitals Leuven, Leuven Cancer Institute, Leuven, Belgium; 4 Department of Oncology, KU Leuven, Leuven, Belgium; 5 Cellular and Molecular Medicine, St. George’s University of London, London, UK; 6 Multidisciplinary Breast Center, University Hospitals Leuven, KU Leuven, Leuven, Belgium; 7 Vesalius Research Center, Vlaams Instituut voor Biotechnologie (VIB), Flanders, Belgium; 8 Department of Oncology, Laboratory for Translational Genetics, KU Leuven, Leuven, Belgium

**Keywords:** Multivariable analysis, Febrile neutropenia, Breast neoplasms, Chemotherapy, Genetics, Single nucleotide polymorphism

## Abstract

**Background:**

Febrile neutropenia (FN) is common in breast cancer patients undergoing chemotherapy. Risk factors for FN have been reported, but risk models that include genetic variability have yet to be described. This study aimed to evaluate the predictive value of patient-related, chemotherapy-related, and genetic risk factors.

**Methods:**

Data from consecutive breast cancer patients receiving chemotherapy with 4–6 cycles of fluorouracil, epirubicin, and cyclophosphamide (FEC) or three cycles of FEC and docetaxel were retrospectively recorded. Multivariable logistic regression was carried out to assess risk of FN during FEC chemotherapy cycles.

**Results:**

Overall, 166 (16.7%) out of 994 patients developed FN. Significant risk factors for FN in any cycle and the first cycle were lower platelet count (OR = 0.78 [0.65; 0.93]) and haemoglobin (OR = 0.81 [0.67; 0.98]) and homozygous carriers of the rs4148350 variant T-allele (OR = 6.7 [1.04; 43.17]) in *MRP1*. Other significant factors for FN in any cycle were higher alanine aminotransferase (OR = 1.02 [1.01; 1.03]), carriers of the rs246221 variant C-allele (OR = 2.0 [1.03; 3.86]) in *MRP1* and the rs351855 variant C-allele (OR = 2.48 [1.13; 5.44]) in *FGFR4*. Lower height (OR = 0.62 [0.41; 0.92]) increased risk of FN in the first cycle.

**Conclusions:**

Both established clinical risk factors and genetic factors predicted FN in breast cancer patients. Prediction was improved by adding genetic information but overall remained limited. Internal validity was satisfactory. Further independent validation is required to confirm these findings.

## Background

Chemotherapy-induced neutropenia (CIN) and febrile neutropenia (FN) are serious and frequent complications in breast cancer patients receiving adjuvant chemotherapy, and they result in hospitalisations [1-3] and chemotherapy dose reductions or delays that impact on treatment outcome and short-term mortality [4]. Adjuvant fluorouracil, epirubicin, and cyclophosphamide (FEC) chemotherapy has an FN risk of between 9% and 14% (low-intermediate risk) [5].

Antibacterial or antifungal prophylaxis has recently been recommended for neutropenic patients expected to have a prolonged low neutrophil count or with other risk factors that favour complications [6]. Prophylaxis with granulocyte colony-stimulating factor (GCSF) in patients at high risk of FN (>20%) is recommended in international guidelines [5,7,8]. For chemotherapy regimens with an intermediate FN risk (10-20%), the European Organisation for Research and Treatment of Cancer (EORTC) GCSF guideline recommends that patient risk factors should also be considered to determine individual risk of FN [5] and the likely benefit of prophylactic GCSF. Therefore, it is important to identify patients at high risk of FN before the initiation of chemotherapy to provide them with appropriate prophylactic measures.

Risk models for the occurrence of CIN [9] and FN [10] in patients with breast cancer have been published. The risk factors identified include older age, lower weight, higher planned dose of chemotherapy, higher number of planned chemotherapy cycles, vascular comorbidity, lower baseline white blood cell count (WBC), lower platelet and neutrophil count, and higher baseline bilirubin. Prior chemotherapy, abnormal liver or renal function, low WBC, higher chemotherapy intensity, and planned delivery were identified as risk factors for neutropenic complications in a prospective US study of patients with different types of cancer [11]. Poor performance status and low lymphocyte and neutrophil counts were risk factors in a European study of solid tumour patients [12], as were tumour stage and number of comorbidities in elderly patients with solid tumours [13].

These risk models of CIN or FN that included patient- or chemotherapy-related factors were reported to be predictive. However, more refined models are necessary to achieve satisfactory performance in independent patient populations that include existing and emerging types of data, including stable genetic factors that are easily measurable, objective, and potentially independent from the inherent viabilities of clinical decision-making. Several studies have assessed the impact of genetic factors on haematological toxicity, but these studies were small in size or limited to only a few candidate genetic factors [14-16].

The objective of this study was to develop risk models for the occurrence of FN in breast cancer patients receiving FEC chemotherapy in any cycle and the first cycle based on a large set of patient-related, chemotherapy-related, and genetic characteristics.

## Methods

### Study population

We retrospectively studied early (i.e., no distant metastases; Stage I-IIIC) breast cancer patients treated between 2000 and 2010 at the Leuven Multidisciplinary Breast Cancer Center of the University Hospitals Leuven, Belgium. Consecutive patients were included if they received either three cycles of neoadjuvant or adjuvant combination chemotherapy consisting of FEC followed by three cycles of docetaxel or four to six cycles of FEC. Patient-related factors (genetics and tumour characteristics) and chemotherapy-related factors were retrospectively recorded in a clinical database. Haematological toxicities included were: FN (defined as an absolute neutrophil count (ANC) < 0.5 × 10^9^/L and a body temperature ≥ 38°C according to the Infectious Diseases Society of America), prolonged grade 4 neutropenia (≥ 5 days), deep neutropenia (< 100/μl), grade 3/4 thrombocytopenia, and grade 3/4 anaemia during FEC chemotherapy cycles. Haematological toxicities that occurred during chemotherapy cycles with docetaxel were not included in the model. Grade 3/4 non-haematological toxicities were also recorded (toxicity grade based on the Common Terminology Criteria for Adverse Events 3.0 [17]). During most of the study period, only primary prevention with GCSF was reimbursed and, therefore, only used in selected patients aged 65 or over. Similarly, secondary use of GCSF was only reimbursed and used if patients had FN in the previous cycle or if deep neutropenia occurred for at least five days (although the latter was not systematically measured during the study period).

The study design and full analysis of single nucleotide polymorphisms (SNPs) have previously been described in detail [18]; however, in the previous analysis the association of SNPs with FN was only adjusted for age, growth factor use, BMI, and planned cycles of chemotherapy. Only those SNPs that have been reported to be associated with haematological toxicity or to play a role in the metabolism of FEC chemotherapy were included in the current study. Logistic regression was performed to describe the association of SNPs with haematological toxicity, adjusted for known predictors of FN risk such as age, growth factor use, and planned number of cycles of chemotherapy. The ethics committee of the University Hospitals Leuven approved the study and all patients included in the study had given written informed consent for collection of genetic samples and for further analyses using this material and associated data.

### Endpoints and predictor variables

The primary endpoint of the study was FN in any cycle, and FN occurring in the first cycle (cycle 1) was the secondary endpoint. The following variables were considered as predictors of FN: planned doses of fluorouracil, epirubicin and cyclophosphamide (FC, 600 mg/m^2^ until August 2004 and 500 mg/m^2^ after this date; epirubicin 100 mg/m^2^), age at diagnosis, height, weight, body mass index (BMI), body surface area (BSA), chemotherapy setting (i.e. adjuvant or neoadjuvant), use of GCSF (information only available on primary or secondary use), planned cycles of FEC chemotherapy, selected SNPs [18], baseline WBC, ANC and platelet count, and other baseline laboratory parameters such as haemoglobin, bilirubin, alanine aminotransferase (ALT), aspartate aminotransferase (AST) and creatinine. Although timing and reasoning of GCSF use were incomplete, its potential impact on the variables included in the final model was assessed for exploratory analysis.

### Statistical analysis

All analyses were performed using Stata/SE version 12.1 (StataCorp LP, College Station, TX, USA). All statistical tests were carried out two-sided at a 5% significance level and 95% confidence intervals (CIs) were obtained.

#### Descriptive and univariable analysis

Binary and categorical data were summarised using frequencies and percentages. Continuous data were reported using means and standard deviations. In the univariable analysis of SNPs, the impact of multiple testing was assessed by separately calculating the false discovery rate (FDR) for each endpoint [19]. Associations between the endpoints and binary or categorical variables were assessed using the chi-squared test or Fisher’s exact test, as appropriate. Continuous variables and their associations with the endpoints were assessed using univariable logistic regression analysis. Variables were further assessed in multivariable logistic regression analysis if a trend was seen in the univariable analysis (*p* ≤ 0.25), as recommended [20]. Linear correlations between potential predictors were assessed by calculating Pearson’s correlation coefficient and monotonic correlations were assessed using Spearman’s rank correlation coefficient. Variables were regarded as being dependent if the correlation coefficient was ≥ 0.7 or the correlation p-value was ≤ 0.05.

#### Multivariable analysis

Multivariable logistic regression analysis was used to assess the joint explanatory value of the candidate variables identified in univariable analysis; variables were included in the final multivariable models if their corresponding *p*-value was ≤ 0.05. Where simultaneous inclusion of dependent variables led to estimation problems (collinearity issues), the variable that explained more of the variability present in the endpoint was finally used. As patient-related and chemotherapy-related factors were already established as risk factors in several previous risk models, these variables were entered into the model first, ordered according to the *p*-value obtained in univariable analysis. SNPs were subsequently added. Interactions between variables were assessed. Model fit was assessed with the Hosmer-Lemeshow [21] goodness-of-fit test. Test characteristics such as specificity (proportion of negatives correctly identified as not having an event), sensitivity (proportion of positives correctly identified as having an event), positive predictive value (PPV, proportion of patients identified to have an event who had an event) and negative predictive value (NPV, proportion of patients identified not to have an event who did not have an event) were obtained. The predictive ability of the final models was assessed by calculating the area under the receiver operating characteristic (ROC; sensitivity over 1-specificity) curve.

To test the internal validity of the final models, nonparametric bootstrapping was performed [22]. Bootstrap estimates of the 95% CIs of the multivariable models were obtained by resampling the data 200 times. The obtained 95% CI estimates of the bootstrap resampling were compared to the 95% CIs calculated by the multivariable logistic regression model.

## Results

### Characteristics of the study group

Of 1,012 patients that received FEC chemotherapy between 2000 and 2010, 18 patients were excluded due to receiving chemotherapy prior to FEC, which may have impacted on FN risk. The majority of 994 eligible patients received adjuvant chemotherapy (*n* = 874, 88.0%); the remainder received neoadjuvant chemotherapy. Most patients received three cycles of combination chemotherapy with FEC followed by three cycles of docetaxel (*n* = 507, 51.0%) or six cycles of FEC (*n* = 405, 40.7%) (Table 1). The most common type of breast cancer was invasive ductal carcinoma (*n* = 823, 82.8%) and patients mostly had grade 2 (*n* = 334, 34.1%) or grade 3 (*n* = 606, 61.9%) tumours. FN occurred in any cycle in 166 (16.7%) patients, of which 107 (10.8%) had FN in the first cycle of FEC chemotherapy. The most common haematological toxicity was prolonged grade 4 neutropenia (*n* = 345, 34.7%). Other haematological toxicities such as grade 3/4 thrombocytopenia and severe bleeding, and grade 3/4 non-haematological toxicities such as diarrhoea, mucositis, and neuropathy were rare (*n* < 10, <1%). Primary prophylactic GCSF (before a CIN or FN event occurred) was given to 15 (1.5%) patients and the majority received no GCSF (*n* = 654, 65.8%). Additional toxicities and other relevant characteristics such as planned number of chemotherapy cycles, tumour stage, and subtype are presented in Table 1. The list of SNPs included in the analyses is shown in Table 2.

**Table 1:** Characteristics of the study population, the tumours, and the administered chemotherapy including toxicities

**Patient characteristics**	**Mean ± standard deviation or frequency (%)**
Age at diagnosis (years) (*n* = 994)	50.4 ± 9.6
Body mass index (kg/m^2^) (*n* = 981)	24.9 ± 4.1
Body surface area (m^2^) (*n* = 993)	1.7 ± 0.1
**Tumour characteristics**	
Tumor status	994 (100)
- Primary tumour	966 (97.2)
- Relapsed tumour	28 (2.8)
Tumour grade^a^	979 (98.5)
- 1	39 (4.0)
- 2	334 (34.1)
- 3	606 (61.9)
Tumour type	994 (100)
- Invasive ductal carcinoma	823 (82.8)
- Invasive lobular carcinoma	103 (10.4)
- Mixed	27 (2.7)
- Others	41 (4.1)
Tumour stage^b^	978 (98.4)
- I	113 (11.5)
- IIA	306 (31.3)
- IIB	245 (25.1)
- IIIA	193 (19.7)
- IIIB	44 (4.5)
- IIIC	77 (7.9)
Receptor status	
- Estrogen receptor positive	683 (68.8)
- Progesterone receptor positive	577 (58.1)
- HER2 positive	205 (20.7)
Subtype^c^	981 (98.7)
- Luminal A	325 (33.1)
- Luminal B HER2-	234 (23.9)
- Luminal B HER2+	121 (12.3)
- HER2-like	84 (8.6)
- Triple negative	217 (22.1)
Nottingham Prognostic Index (NPI)^d^ (*n* = 757)	5.0 ± 0.9
**Chemotherapy characteristics**	
Chemotherapy setting	994 (100)
- Adjuvant	874 (87.9)
- Neoadjuvant	120 (12.1)
Planned cycles of FEC chemotherapy	994 (100)
- 3 cycles FEC	559 (56.2)
- 4 or 5 cycles FEC	2 (0.2)
- 6 cycles FEC	433 (43.6)
Relative dose intensity (RDI) (*n* = 994)	0.96 ± 0.1
Growth factor use	994 (100)
- Primary	15 (1.5)
- Secondary	325 (32.7)
- None	654 (65.8)
**Baseline laboratory parameters**	
White blood cell count (10^9^/L) (*n* = 985)	7.2 ± 2.0
Absolute neutrophil count (10^9^/L) (*n* = 937)	4.4 ± 1.6
Haemoglobin (g/dl) (*n* = 989)	13.3 ± 1.0
Platelets (10^9^/L) (*n* = 985)	275.4 ± 65.1
Total bilirubin (mg/dl) (*n* = 915)	0.4 ± 0.2
Creatinine (mg/dl) (*n* = 957)	0.8 ± 0.1
Alanine aminotransferase (U/L) (*n* = 955)	23.3 ± 15.3
Aspartate aminotransferase (U/L) (*n* = 955)	21.9 ± 11.1
**FEC chemotherapy toxicities**	
Febrile neutropenia	166 (16.7)
- Febrile neutropenia in first cycle	107 (10.7)
Prolonged (≥ 5 days) grade 4 neutropenia	345 (34.7)
Deep neutropenia (< 100/μl)	93 (9.4)
Other grade 3–4 toxicities	46 (4.6)

FEC, fluorouracil, epirubicin and cyclophosphamide; HER2, human epidermal growth factor receptor 2.

^a^According to the Ellis and Elston grading system [23].

^b^According to the TNM classification [24].

^c^According to Brouckaert et al. [25].

^d^According to Lee et al. [26].

**Table 2:** List of included single nucleotide polymorphisms (SNPs), and their frequencies (percentages)

	**Genotype**					
**Gene**	**n**	**GG**	**GA**	**AA**	**CC**	**CA**
		**n (%)**	**n (%)**	**n (%)**	**n (%)**	**n (%)**
ABCC2/MRP2rs8187710	954	842 (88.3)	110 (11.5)	2 (0.2)		
ABCG2/BRCPrs2231137	955	888 (93.0)	67 (7.0)			
CYP2B6rs2279343	910	57 (6.2)	382 (42.0)	471 (51.8)		
CYP2C8rs72558196	960			960 (100)		
CYP2C9rs1057910	954			853 (89.4)	3 (0.3)	98 (10.3)
CYP2C19rs4244285	946	652 (68.9)	266 (28.1)	28 (3.0)		
CYP2C19rs4986893	960	960 (100)				
CYP3A4rs2740574	955	2 (0.2)	57 (6)	896 (93.8)		
CYP3A4rs55785340	957			957 (100)		
CYP3A5rs776746	959	834 (87.0)	118 (12.3)	7 (0.7)		
DPYDrs1801159	960	47 (4.9)	267 (27.8)	646 (67.3)		
DPYDrs3918290	949	945 (99.6)	4 (0.4)			
DPYDrs1801160	957	853 (89.1)	96 (10.0)	8 (0.9)		
GSTA1rs3957357	938	329 (35.1)	441 (47.0)	168 (17.9)		
GSTP1rs1695	959	118 (12.3)	452 (47.1)	389 (40.6)		
MRP1rs1883112	956	295 (30.9)	485 (50.7)	176 (18.4)		
MRP1rs7853758	952	701 (73.6)	231 (24.3)	20 (2.1)		
MTHFRrs1801131	951			446 (46.9)	92 (9.7)	413 (43.4)
UGT2B7rs12233719	949	949 (100)				
UGT2B7rs7662029	955	210 (22.0)	473 (49.5)	272 (28.5)		
XPD/ERCC2rs1799793	954	412 (43.2)	429 (45.0)	113 (11.8)		
XRCC1rs25489	954	875 (91.7)	77 (8.1)	2 (0.2)		
XRCC3rs861534	949	357 (37.6)	441 (46.5)	151 (15.9)		
**Gene**	**n**	**TT**	**CC**	**CT**	**AA**	**TA**
		**n (%)**	**n (%)**	**n (%)**	**n (%)**	**n (%)**
ABCC2/MRP2rs17222723	951	843 (88.6)			2 (0.2)	106 (11.2)
ABCC2/MRP2rs2804402	935	297 (31.8)	185 (19.8)	453 (48.4)		
CYP2B6rs8192709	927		846 (91.3)	87 (8.7)		
CYP2C8rs10509681	960	740 (77.1)	12 (1.2)	208 (21.7)		
CYP2C9rs1799853	957	15 (1.6)	712 (74.4)	230 (24)		
CYP3A4rs4986910	959	938 (97.8)		21 (2.2)		
DPYDrs1801265	960	635 (66.1)	46 (4.8)	279 (29.1)		
FGFR4rs351855	954	88 (9.2)	461 (48.3)	405 (42.5)		
GSTP1rs1138272	952	6 (0.6)	778 (81.7)	168 (17.7)		
MDRI/ABCB1rs1045642	914	265 (29.0)	208 (22.8)	441 (48.2)		
MRP1rs13058338	949	482 (50.8)			67 (7.1)	400 (42.1)
MRP1rs246221	956	462 (48.3)	71 (7.4)	423 (44.3)		
MRP1rs3743527	930	13 (1.4)	562 (60.4)	355 (38.2)		
MRP1rs4673	954	115 (12.0)	406 (42.6)	433 (45.4)		
MTHFRrs1801133	959	121 (12.6)	401 (41.8)	437 (45.6)		
NQO1rs1800566	958	35 (3.6)	605 (63.2)	318 (33.2)		
UGT2B7rs7439366	955	272 (28.5)	210 (22.0)	473 (49.5)		
UGT2B7rs7668282	954	940 (98.5)	1 (0.1)	13 (1.4)		
**Gene**	**n**	**GG**	**GT**	**TT**	**CC**	**CG**
		**n (%)**	**n (%)**	**n (%)**	**n (%)**	**n (%)**
ALDH3A1rs2228100	934	67 (7.2)			554 (59.3)	313 (33.5)
CYP2B6rs3745274	954	535 (56.1)	365 (38.2)	54 (5.7)		
GPX4rs757229	940	263 (28.0)			212 (22.5)	465 (49.5)
MRP1rs4148350	957	847 (88.5)	105 (11.0)	5 (0.5)		
MRP1rs45511401	960	847 (88.2)	109 (11.4)	4 (0.4)		
UGT2B7rs3924194	954	19 (2.0%)			712 (74.6)	223 (23.4)
XPD/ERCC2rs13181	951	116 (12.2)	449 (47.2)	386 (40.6)		
**Gene**	**n**	**GG**	**GT**	**TT**	**GA**	**TA**
		**n (%)**	**n (%)**	**n (%)**	**n (%)**	**n (%)**
MDRI/ABCB1rs2032582	948	283 (29.9)	445 (46.9)	185 (19.5)	23 (2.4)	12 (1.3)
TYMSrs11280056	918	AAGTTA 442 (48.2)		AAGTTA.DEL 394 (42.9)		DEL 82 (8.9)

### Univariable analysis

All candidate predictors (p ≤ 0.25) for FN in any cycle and in cycle 1 are shown in Table 3. Patient-related factors (genetics, laboratory parameters, etc.) and chemotherapy-related factors fulfilled the inclusion criteria for the multivariable analysis. The number of planned FEC cycles, WBC, ANC, platelet count, and haemoglobin were significantly associated with FN in any cycle and cycle 1 (p ≤ 0.05). SNPs significantly associated with FN in any cycle and cycle 1 were the rs4148350, rs45511401, and rs246221 variants in *MRP1* (multidrug resistance-associated protein 1)*.* The FDR for associated SNPs for any cycle FN was 0.47 and 0.33 for cycle 1 FN. There were no correlations between SNPs included in the final model and patient-related or chemotherapy-related factors.

**Table 3:** Candidate predictors from univariable analysis

	**FN in any cycle**		**FN in cycle 1**	
**Variable**	**OR (95% CI)**	**p-value**	**OR (95% CI)**	**p-value**
Platelets (10^9^/L, per 10 units change)	0.96 (0.93; 0.98)	0.002	0.95 (0.92; 0.99)	0.005
ANC (10^9^/L)	0.87 (0.77; 0.98)	0.023	0.86 (0.74; 1.00)	0.046
ALT (U/L, per 10 units change)	1.12 (1.02; 1.23)	0.024	-	-
WBC (10^9^/L)	0.90 (0.83; 0.99)	0.032	0.88 (0.79; 0.99)	0.028
Height (cm)	-	-	1.03 (1.00; 1.07)	0.043
Haemoglobin (g/dl)	0.87 (0.73; 1.02)	0.094	0.80 (0.66; 0.98)	0.030
Planned cycles FEC (6 vs. 3 cycles)	1.09 (0.98; 1.22)	0.129	-	-
AST^a^ (U/L, per 10 units change)	1.09 (0.95; 1.24)	0.210	-	-
BSA (m2)	-	-	2.44 (0.59; 10.03)	0.217
Creatinin (mg/dl)	2.04 (0.66; 6.33)	0.219	-	-
Planned dose of epirubicin (100 mg/m^2^)	-	-	1.01 (0.99; 1.02)	0.217
MRP1rs4148350		0.000		0.004
- GT vs. GG	1.82 (1.12; 2.94)	0.015	2.09 (1.21; 3.61)	0.008
- TT vs. GG	22.06 (2.45; 198.96)	0.006	6.30 (1.04; 38.28)	0.045
MRP1rs45511401^b^		0.000		0.004
- GT vs. GG	1.80 (1.12; 2.89)	0.015	1.82 (1.05; 3.17)	0.034
- TT vs. GG	16.40 (1.69; 158.84)	0.016	9.20 (1.28; 66.20)	0.027
MRP1rs246221		0.004		0.039
- TT vs. CC	0.47 (0.25; 0.86)	0.014	0.49 (0.24; 1.00)	0.053
- TC vs. CC	0.80 (0.44; 1.45)	0.459	0.80 (0.40; 1.61)	0.530
FGFR4rs351855		0.098	-	-
- CT vs. CC	1.25 (0.88; 1.77)	0.216		
- TT vs. CC	0.60 (0.29; 1.24)	0.166		
CYP3A4rs4986910		0.171	-	-
- TC vs. TT	0.24 (0.03; 1.84)			
XRCC3rs861534		0.130		0.044
- GG vs. AA	1.25 (0.76; 2.07)	0.381	1.73 (0.91; 3.29)	0.095
- GA vs. AA	0.86 (0.52; 1.42)	0.544	1.03 (0.53; 1.99)	0.930
TYMSrs11280056		0.114	-	-
- AAGTTA.DEL vs. AAGTTA	0.88 (0.60; 1.27)	0.486		
- DEL vs. AAGTTA	1.60 (0.91; 2.82)	0.100		
GSTP1rs1695		0.228	-	-
- AG vs. AA	0.75 (0.53; 1.08)	0.124		
- GG vs. AA	0.70 (0.40; 1.25)	0.231		
GSTA1rs3957357	-	-		0.163
- GG vs. AA			0.95 (0.49; 1.83)	0.875
- GA vs. AA			1.45 (0.80; 2.65)	0.223
ALDH3A1rs2228100	-	-		0.188
- GG vs. CC			1.86 (0.92; 3.76)	0.086
- GC vs. CC			1.27 (0.81; 1.98)	0.297
MRP1rs1883112	-	-		0.187
- AG vs. AA			0.87 (0.52; 1.46)	0.594
- GG vs. AA			0.59 (0.32; 1.08)	0.087
UGT2B7rs7439366	-	-		0.204
- TT vs. CC			1.08 (0.57; 2.04)	0.813
- TC vs. CC			1.52 (0.87; 2.65)	0.139
UGT2B7rs7662029	-	-		0.204
- GG vs. AA			0.93 (0.49; 1.75)	0.813
- GA vs. AA			1.41 (0.86; 2.31)	0.174

ALT, alanine aminotransferase; ANC, absolute neutrophil count; AST, aspartate aminotransferase; BMI, body mass index; BSA, body surface area; CI, confidence interval; FEC, fluorouracil, epirubicin and cyclophosphamide; FN, febrile neutropenia; WBC, white blood cell count.

Odds ratios and 95% confidence intervals are reported per 1 unit change if not otherwise indicated.

^a^Highly correlated with alanine aminotransferase (Pearson’s correlation coefficient 0.76) and not included in multivariable analysis.

^b^Highly correlated with MRP1rs4148350 (Spearman correlation coefficient 0.81) and not included in multivariable analysis.

### Risk factors of febrile neutropenia in any cycle

Multivariable regression identified the following factors to be significantly associated with a higher occurrence of FN: lower platelet count and lower haemoglobin at baseline, higher ALT, and the following SNPs: rs4148350 and rs246221 in *MRP1* and rs351855 in *FGFR4* (fibroblast growth factor receptor 4) (Table 4). Homozygous carriers of the rs4148350 T-allele had a higher risk of FN than carriers of the homozygous or heterozygous G-allele (FN risk of 80% versus 15% or 25%). For rs246221, homozygous carriers of the T-allele variant had a lower risk of FN than carriers with at least one C-allele (FN risk of 13% versus 20% or 24%). Patients with the TT genotype of rs351855 were protected against FN compared to patients carrying at least one C-allele (FN risk of 10% versus 19% or 16%).

**Table 4:** Logistic regression models for febrile neutropenia occurrence in any cycle and the first cycle of chemotherapy

**Determinant**	**FN in any cycle (*n*** **= 910)**		**FN in cycle 1 (*n*** **= 937)**	
	**Odds ratio (95% CI)**	***p*-value**	**Odds ratio (95% CI)**	***p*-value**
Platelets (10^9^/L, per 10 units change)	0.952 (0.923; 0.981)	0.001	0.951 (0.917; 0.985)	0.006
Hb (g/dl)	0.812 (0.673; 0.978)	0.029	0.001 (<0.001; 0.194)	0.009
Height (cm)	-	-	0.617 (0.414; 0.919)	0.018
Interaction (height and Hb)^a^	-	-	1.040 (1.008; 1.072)	0.012
ALT (U/L, per 10 unit change)	1.173 (1.056; 1.303)	0.003	-	-
MRP1rs4148350		0.019		0.006
- GT^b^ vs. GG	1.494 (0.890; 2.507)	0.129	2.149 (1.226; 3.768)	0.008
- TT^c^ vs. GG	17.13 (1.72; 170.90)	0.016	6.696 (1.039; 43.167)	0.046
MRP1rs246221		0.023	-	-
- TT^d^ vs. CC	0.501 (0.259; 0.969)	0.040		
- TC vs. CC	0.805 (0.423; 1.533)	0.510		
FGFR4rs351855		0.062	-	-
-CT vs. CC	1.253 (0.862; 1.821)	0.238		
-TT^e^ vs. CC	0.505 (0.230; 1.113)	0.090		

ALT, alanine aminotransferase; CI, Confidence interval; FN, febrile neutropenia; HB, haemoglobin.

Odds ratios and 95% confidence intervals are reported per 1 unit change if not otherwise indicated.

^a^Did not affect the odds ratio of the other main effects of the regression model.

^b^105/957 (11.0%) patients are carriers of the GT genotype and 19 (18.1%) out of those 105 patients had febrile neutropenia in cycle 1 of chemotherapy.

^c^5/957 (0.5%) patients are homozygous carriers of the T-allele and 4 (80%) out of those 5 patients had febrile neutropenia in any cycle of chemotherapy and 2 (40%) had febrile neutropenia in cycle 1.

^d^462/956 (48.3%) patients are homozygous carriers of the T-allele and 59 (12.8%) out of those 462 patients had febrile neutropenia in any cycle of chemotherapy.

^e^88/954 (9.2%) patients are homozygous carriers ot the T-allele and 9 (10.2%) out of those 88 patients had febrile neutropenia in any cycle of chemotherapy.

**Figure 1:**
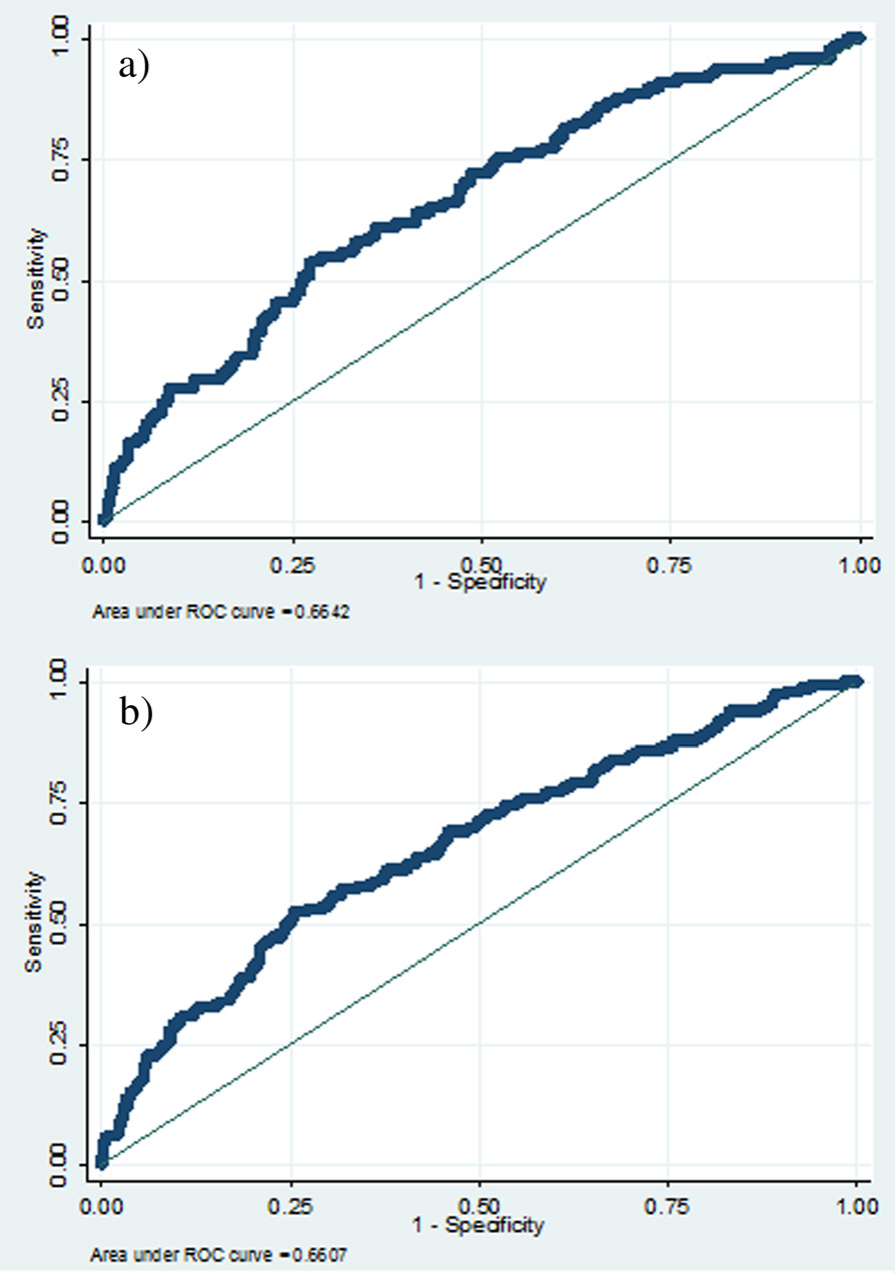
**Receiver operating characteristic curve for febrile neutropenia occurrence in a) any cycle and b) cycle 1 of chemotherapy.** ROC, receiver operating characteristic. *bysecting line indicates a predictiove ability that is no better than chance (ROC = 0.5).

The area under the ROC curve was 0.661 (CI 0.629-0.691), as shown in Figure 1a: a value of 1 would denote perfect discrimination and 0.5 discrimination no better than chance. Overall, 864 of 910 patients (84.0%) were correctly classified by the logistic regression model at a predicted probability cut-off of 0.5; six out of 150 having FN and 758 out of 760 not having FN. Sensitivity was very low (4.0%) compared to specificity (99.7%). NPV and PPV were similar; the proportion of patients correctly identified not to have FN was 84.0% and the proportion of patients correctly identified to have FN was 75.0%. When the optimal cut-off of the model was used (i.e., predicted probability of 0.1609, where sensitivity and specificity were almost identical at 61.3%), the model correctly classified 61.2% of the patients and PPV and NPV were 23.8% and 88.9%, respectively. Internal validity of the FN in any cycle model was satisfactory; the 95% CIs of the bootstrap resampling were similar to the 95% CIs calculated by the multivariable logistic regression model.

### Risk factors of febrile neutropenia in cycle 1

Lower platelet count, haemoglobin at baseline, and lower patient height were significantly associated with a higher risk of FN in cycle 1 (Table 4). The SNP found to be significantly associated with FN in cycle 1 was rs4148350 in *MRP1*. For rs4148350, homozygous carriers of the T-allele had a higher risk of FN in cycle 1 than carriers of the homozygous or heterozygous G-allele (FN risk of 40% versus 10% or 18%). We found a statistically significant interaction between haemoglobin and height that increased the protective effect of higher haemoglobin and increased height but did not affect the other main effects of the model.

The area under the ROC curve was 0.664 (CI 0.633-0.694), as presented in Figure 1b. At a probability cut-off of 0.5, one out of 98 patients was correctly classified having FN in cycle 1 and all 839 patients without FN in cycle 1 were correctly classified not having FN (overall, 89.7% correct classifications). Sensitivity was very low (1.0%); specificity was 100%, PPV was 100%, and NPV was 89.6%. At the optimal probability cut-off for the model (0.1041), 61.5% of the patients were correctly classified, sensitivity and specificity were 61%, PPV was 15.7%, and NPV was 93.1%. The 95% CIs of the bootstrap resampling were similar to the 95% CIs calculated by the multivariable logistic regression model, which supports the internal validity of the FN in the first cycle model.

## Discussion

In this population of early breast cancer patients seen in routine clinical practice at a tertiary referral centre, we identified a set of genetic factors, in addition to patient-related and chemotherapy-related factors, that predict occurrence of FN in any cycle or the first cycle of chemotherapy. Significant predictors of a higher risk of FN in any cycle and in cycle one were: lower baseline platelet count, lower baseline haemoglobin, and carriers of the rs4148350 T-allele variant in *MRP1*, especially homozygous T-allele carriers. Patients with lower ALT and homozygous carriers of the rs246221 variant T-allele in *MRP1* and rs351855 variant T-allele in *FGFR4* had a lower risk of FN occurrence. Although the predictive ability of the models was improved by including genetic factors, the overall predictive ability remained poor. Genetic effects were stable and FN occurrence was very high in patients with specific SNP allele variants.

The observed effects of lower baseline platelet count and haemoglobin are consistent with previous reports. Baseline platelet count has been shown to differ between cancer patients with mild and severe haematological toxicity [16], and low haemoglobin has been mentioned as possible risk factor for FN [27] and survival [28]. In the model of FN occurrence in any cycle, higher baseline ALT was significantly associated with FN but not baseline bilirubin [9,29]. Both measures are indicators of liver function and since the liver detoxifies drugs like epirubicin [30], impaired liver function may be an important risk factor for FN occurrence in patients receiving chemotherapy with epirubicin. A predictive role for WBC or ANC in CIN and FN occurrence in cancer patients receiving chemotherapy has been described in other studies [9-12], but could not be confirmed in our models. Most SNPs previously associated with FN occurrence [18] and reported to be involved in anthracycline-induced cardiotoxicity [31-33] were confirmed in the multivariable analysis. The SNP rs45511401 was not included in the multivariable regression model as it was highly correlated with rs4148350, and the latter variant explained the model variability slightly better. There were no correlations between SNPs included in the final model and patient- or chemotherapy-related factors.

International guidelines [5,7,8] and the literature [9,12] report age, planned dose intensity, and planned number of chemotherapy cycles to be important risk factors for CIN and FN during chemotherapy. These risk factors could not be confirmed in our models. Patient-specific approaches to clinical management were not recorded in detail in this study and might therefore have masked the effect of age on FN occurrence. In addition, the exact cycle of FN occurrence was not available after the first cycle. Factors previously reported to protect against CIN and FN in any cycle of chemotherapy, such as dose reductions, dose delays, or growth factor use before an event occurred, could not be investigated since the details, reasons, and timing information were not available and only 15 out of 994 patients received primary prophylaxis with GCSF, mainly due to reimbursement criteria.

The apparent predictive ability, i.e., the predictive ability assessed in the ‘training’ dataset used to develop the models, was lower than in previously published models of CIN or FN occurrence in other cancers [9,11,34]. In these models, sensitivity and specificity at the optimal predicted probability cut-off was about 70% or higher, but in this study it remained below 70%. As commonly seen in models of FN occurrence, the NPV (≥ 90%) was much higher than the PPV because FN incidence is often around 20%; this implies an NPV of around 80% for simply assuming that FN does not occur in any patient. The areas under the ROC curves were relatively low but significantly higher than 0.5, the value indicating no predictive ability. In other words, the models allowed partial discrimination of patients at low or high risk of FN. Including genetic risk factors improved the models but absolute predictive ability remained rather low. The effects of the SNPs were stable and FN occurrence was very high in patients with specific, sometimes rare, SNP allele variants. In terms of clinical implications, genetic testing might help to identify a small proportion of patients at very high risk of FN who can be targeted with prophylactic measures. For the majority of patients, the current models do not reliably identify patients that will develop FN, but they do delineate patients who are unlikely to develop FN. This is clinically relevant since patients at low risk of FN probably do not need primary GCSF prophylaxis or nadir assessment, while the high-risk group is unpredictable and might need more extensive preventive measures or follow-up.

The performance of any model tends to be highest in the training dataset. The results obtained with bootstrap resampling supported the internal validity of the FN in any cycle and the FN in first cycle models. The predictive ability of the models has yet to be tested in an entirely independent population, where model performance is usually lower. Before risk models are put to clinical use, true external validation is essential [35,36]. Another limitation of this study is the retrospective design; no detailed information was available on patient management in clinical practice, which is known to influence the risk of FN occurrence, and the reasons and timing of dose reductions and dose delays were not available. FN occurrence was not assessed according to chemotherapy cycle beyond the first cycle. GCSF was only administered to 15 patients before an event occurred due to stringent reimbursement criteria. Hence, the impact of GCSF on FN occurrence was difficult to assess.

To the best of our knowledge, this is the first study of risk of FN in the first and any cycle of chemotherapy in patients with early breast cancer that combined a set of patient- and chemotherapy-related factors with a large set of SNPs. Further validation studies are needed to confirm our findings, which should ideally be prospectively designed, sufficiently powered, and measure all possible predictors of FN occurrence reported in the literature. Approaches to clinical management that are measurable and known to influence the risk of FN occurrence, such as dose modifications or growth factor use before an FN event occurred, should be included. Information on SNPs should be available for as many patients as possible and the frequencies of possible genotypes of one SNP should be similar. Validated genetic factors have the potential to become reliable predictors of FN occurrence. The specific SNPs that were assessed in this study are independent from clinical decision-making and therefore less likely to be confounded by clinical practice.

## Conclusions

We have identified a set of chemotherapy-related, patient-related, and genetic risk factors that predict occurrence of FN in the first and any cycle of chemotherapy in a large cohort of early breast cancer patients. Genetic effects in the models improved the predictive ability, but the overall predictive ability of the models remained poor. FN occurrence was very high in patients with specific SNP allele variants. Up-front genetic testing might be helpful to identify a limited group of very high-risk patients. Further independent validation is required to develop risk models that include genetic predictors of FN occurrence and can be used to personalise care.

## Abbreviations

ALT: Alanine aminotransferase; ANC: Absolute neutrophil count; AST: Aspartate aminotransferase; BMI: Body mass index; BSA: Body surface area; CI: Confidence interval; CIN: Chemotherapy-induced neutropenia; EORTC: European Organisation for Research and Treatment of Cancer; FDR: False discovery rate; FEC: Fluorouracil, epirubicin and cyclophosphamide; FN: Febrile neutropenia; FGFR: Fibroblast growth factor receptor; GCSF: Granulocyte colony-stimulating factor; MRP1: Multidrug resistance-associated protein 1; NPI: Nottingham Prognostic Index; NPV: Negative predictive value; OR: Odds ratio; PPV: Positive predictive value; ROC: Receiver operating characteristic; SNP: Single nucleotide polymorphism; WBC: White blood cell count.

## Competing interests

AMP receives research funding from Amgen via the employing institution. HW has received lecture fees from Amgen. MS receives research funding from Amgen via the employing institution and has served on advisory boards for Amgen. RPe is on the speaker bureau for Amgen. All the other authors declare no conflicts of interest related to this article.

## Authors’ contributions

AMP was responsible for analysis and data interpretation and drafted the manuscript. CV was responsible for data collection and data interpretation and helped to draft the manuscript. RPa participated in study design and data collection. ASD participated in study design and analysis and was responsible for data collection and management. RPe participated in data analysis and data interpretation. SH, PN, and DL participated in study design, data collection and data interpretation. TDS participated in data analysis and data interpretation. MS supervised data analysis and participated in data interpretation. HW was responsible for study design, participated in data collection, and the interpretation of data. MS and HW share last authorship. All authors reviewed the manuscript and read and approved the final manuscript.

## Pre-publication history

The pre-publication history for this paper can be accessed here:

http://www.biomedcentral.com/1471-2407/14/201/prepub
